# TCR Coexpression Signature Predicts Immunotherapy Resistance in NSCLC

**DOI:** 10.3389/fphar.2022.875149

**Published:** 2022-05-04

**Authors:** Yuntao Wang, Yi Liu, Xiaohua Li, Weiming Li, Zhihong Xue, Xiaoqian He, Weijie Xiong, Lang He, Yifeng Bai

**Affiliations:** ^1^ Department of Oncology, The Fifth People’s Hospital Affiliated to Chengdu University of Traditional Chinese Medicine the Second Clinical Medical College, Chengdu, China; ^2^ Wenjiang District People’s Hospital of Chengdu City, Chengdu, China; ^3^ Department of Respiratory and Critical Care Medicine, Sixth People’s Hospital of Chengdu, Chengdu, China; ^4^ Department of Oncology, Sichuan Provincial People’s Hospital, University of Electronic Science and Technology of China, Chengdu, China

**Keywords:** ICIs, tumor immune microenvironment, NSCLC, biomarker, TCR—T cell receptor

## Abstract

**Background**: Lung cancer has the highest morbidity and mortality rate among types of malignant tumors, and as such, research into prolonging the survival time of patients is vital. The emergence of immune checkpoint inhibitors (ICIs) has greatly improved the survival of patients with non-small cell lung cancer (NSCLC), however, the lack of effective biomarkers to predict the prognosis of immunotherapy has made it difficult to maximize the benefits. T cell receptor (TCR) is one of the most important components for recognizing tumor cells, and with this study we aim to clarify the relationship between TCR coexpression and the prognosis of NSCLC patients receiving immunotherapy.

**Methods**: Univariate COX regression, logistics regression, and KM survival analysis were used to evaluate the relationship between TCR coexpression and the prognosis of immunotherapy. Additionally, CIBERSORT, Gene Set Enrichment Analysis (GSEA), and single-sample GSEA (ssGSEA) algorithms were used to evaluate the tumor immune microenvironment (TIME) of NSCLC patients.

**Results**: Univariate Cox regression analysis showed that the TCR coexpression signature can be used as a clinical prognostic indicator for NSCLC patients receiving immunotherapy (*p* = 0.0205). In addition, those in the NSCLC group with a high TCR coexpression signature had significantly improved progression-free survival (PFS) (*p* = 0.014). In the ICI treatment cohort (GSE35640). In addition, there was a high infiltration of CD8+T cells, activated memory CD4+T cells, and M1 macrophages in the TIME of those with a high TCR coexpression signature. The results of pathway enrichment analysis showed that patients with a high TCR coexpression signature had significantly activated signal pathways such as lymphocyte proliferation and activation, chemokine binding, and inflammatory cytokine production. Also, we found that patients with a high TCR coexpression signature had an elevated T cell inflammation gene expression profile (GEP).

**Conclusion**: We show that the TCR coexpression signature may be useful as a new biomarker for the prognosis of NSCLC patients undergoing immunotherapy, with high signatures indicating better treatment response. Additionally, we found that patients with a high TCR coexpression signature had tumor immune microenvironments with beneficial anti-tumor characteristics.

## Introduction

Lung cancer currently has the highest morbidity (11.6%) and mortality (18.4%) among all tumors (Bray et al., 2018). About 75% are advanced (stage III–IV) at the time of diagnosis, and the 5-years survival rate is less than 20%. Non-small cell lung cancer (NSCLC) is the most common, accounting for 80–85% of all lung cancers, and about 57% of patients with advanced NSCLC have distant metastasis at the time of diagnosis (Zappa and Mousa, 2016; Guan et al., 2019). Chemotherapy, targeted therapy, and anti-angiogenic drugs have become the cornerstone of treatment for these patients, but in recent years, the emergence of immune checkpoint inhibitors (ICIs) has changed the approach to NSCLC treatment. According to the literature, the 5-years survival rate of NSCLC patients receiving multi-line therapy that includes immunotherapy treatment can reach 16% (Gettinger et al., 2018). For a non-selective population, the objective response rate (ORR) of ICIs administered without other drugs has been measured at 19–22%. In order to further optimize the benefits of immunotherapy, it is necessary to find biomarkers suitable for predicting the curative effect.

With the rapid development of research in this field, many prognostic markers relating to immune checkpoint blockade (ICB) therapy have been found (Lin et al., 2021). The existing markers for predicting the efficacy of immunotherapy, however, have their limitations. The application of PD-L1 is limited by the variations of time, tumor heterogeneity, and differences in detection method thresholds (Velcheti et al., 2014; McLaughlin et al., 2016; Hui et al., 2017; Mok et al., 2019) The use of tumor mutational burden (TMB) is limited due to the complexity and high cost of whole exon sequencing (WES) (Luo et al., 2019), with the biggest obstacle being the complicated threshold standard, which is difficult to determine (Gandara et al., 2018). Additionally, in regard to NSCLC, although some studies have shown that high microsatellite instability (MSI-H) is related to the efficacy of ICB (Le et al., 2015; Goodman et al., 2017; Prelaj et al., 2019; Niu et al., 2020; Huang et al., 2021; Zhang et al., 2021), MSI-H is very rare in lung cancer. Whether MSI-H can be used as an effective immunotherapy marker for NSCLC patients remains unverified. Therefore, there is still a need to investigate biomarkers and establish models that predict the curative effect to further screen for the patients who would benefit most from the treatment.

Immune repertoire is defined as the total number of T cells and B cells with functional diversity within an individual’s circulatory system at any given time, and is a measure of the diversity and specificity of the individual’s immune state (Looney et al., 2019). T lymphocytes recognize new tumor antigens and proliferate via the T cell antigen receptor (TCR), which is the key process in activating the host immune response against cancer cells. As these T cells carry TCR to recognize and eliminate tumor cells, TCR expression plays an important role in immunotherapy (Looney et al., 2019; Morita et al., 2020). Many studies have confirmed that the characteristics of the baseline TCR repertoire are related to the curative effect of therapy (Manuel et al., 2012; Robins, 2013; Robert et al., 2014; Postow et al., 2015). For example, low baseline T-cell diversity in the peripheral blood of breast cancer patients undergoing chemotherapy has been linked with poor prognosis (Manuel et al., 2012). According to Postow’s research, after CTLA-4 was used, the increased TCR diversity at baseline was related to an increase in efficacy and benefits (Postow et al., 2015). In addition, studies have shown that patients with low T cell diversity in peripheral blood can receive great benefit from anti-PD-1 treatment (Hogan et al., 2019). Currently, there is no research in the literature on the relationship between TCR coexpression signature and the efficacy of NSCLC after receiving ICB. Therefore, in this study we explore and verify the role of TCR (specifically the TCR coexpression signature) in predicting the prognosis of NSCLC patients after immunotherapy at the level of the TIME. With these results, we aim to better identify the population who may benefit most from ICB therapy.

## Methods

### Collection of Immunotherapy Cohort and TCGA Cohort Data

We downloaded an NSCLC cohort published by Hwang and colleagues on anti-PD-1 from the GEO database, which we named ICI-NSCLC (GSE136961) (Hwang et al., 2020). This cohort includes clinical prognosis and expression data for patients who received immunotherapy. In addition, we downloaded the expression and clinical data of TCGA-LUAD and TCGA-LUSC cohorts from the GDC database using the R package named TCGAbiolinks (Colaprico et al., 2016). In order to better study the population of NSCLC, we combined the TCGA-LUAD and TCGA-LUSC cohorts and named it the TCGA-NSCLC cohort. Because there were very few NSCLC cohorts with both ICI treatment data and expression data, we collected a melanoma cohort with ICI treatment from the GEO database and named it ICI-Melanoma (GSE35640) (Ulloa-Montoya et al., 2013). We also obtained an open-source bladder cancer cohort treated with ICIs from a published article by Mariathasan and his colleagues, which we designated ICI-BLCA (Mariathasan et al.) (Mariathasan et al., 2018; Zhou et al., 2021).

### Calculation and Grouping of TCR Coexpression Signatures

According to the gene set definition in the expression data published by Hwang and his colleagues, we used the ssGSEA algorithm (Reas et al., 2007) and the R package named Gene Set Variation Analysis (GSVA) (Hänzelmann et al., 2013) to analyze each patient in the ICI-NSCLC (GSE136961), ICI-Melanoma (GSE35640), ICI-BLCA (Mariathan et al.), and TCGA-NSCLC cohorts. For each cohort, we divided the patients into high and low groups according to the median value of TCR coexpression signatures of all patients in each cohort. The gene set of TCR coexpression signature was detailed in Supplementary Table S1.

### Tumor Immune Microenvironment Analysis

First, we uploaded the expression data of each cohort to the CIBERSORT webtool (Chen et al., 2018), selected LM22, set the number of iterations to 1,000, and analyzed the results. From this, we were able to measure the prevalence of 22 types of immune cells for each patient in each cohort. Secondly, as one of the important targets of ICIs, data on immune checkpoint molecules were obtained from a published study for comparison (Rooney et al., 2015). Data on the genes and molecules that play a very important role in TIME was also obtained from published studies (Rooney et al., 2015; Thorsson et al., 2018). Using this data, we were able to compare the abundance of immune cells, the expression of immune checkpoint molecules, and the expression of immune-related genes among the high and low groups to determine which elements played a vital role.

### Pathway Activity Analysis

We performed a difference analysis on the expression data for each cohort using the R package named Limma (Ritchie et al., 2015), and we used the results as the input file for gene set enrichment analysis (GSEA). We then used the R package named ClusterProfiler to analyze the enrichment of gene sets in the GO-BP, GO-CC, GO-MF, KEGG, and REACTOME databases according to the ranked list (including ENTREZID and logFC) (Subramanian et al., 2005). In addition, we used the R package named GSVA to analyze the ssGSEA of gene sets from the Molecular Signatures Database (MSigDB) (https://www.gsea-msigdb.org/gsea/msigdb/genesets.jsp). By obtaining the ssGSEA score for each patient, we were able to further compare the activity differences between the high and low groups on the same pathway.

### Statistical Analysis

For continuous variables such as the abundance of immune cells, the expression of genes, and ssGSEA score, we used the Mann-Whitney U test to compare differences between the high and low groups. To study the predictive effect of TCR coexpression on the prognosis of immunotherapy, we used univariate COX regression, Kaplan-Meier (KM) regression, and logistics regression analysis. For the KM analysis, log-rank P was used to evaluate statistical significance. All the analyses in this study were conducted on R software (Version. 3.7). Statistical significance was evaluated by *p* value, with *p* < 0.05 regarded as having statistical difference and being bilateral.

## Results

### High TCR Coexpression Signature Indicated Better Prognosis and Response to Immunotherapy

**FIGURE 1:**
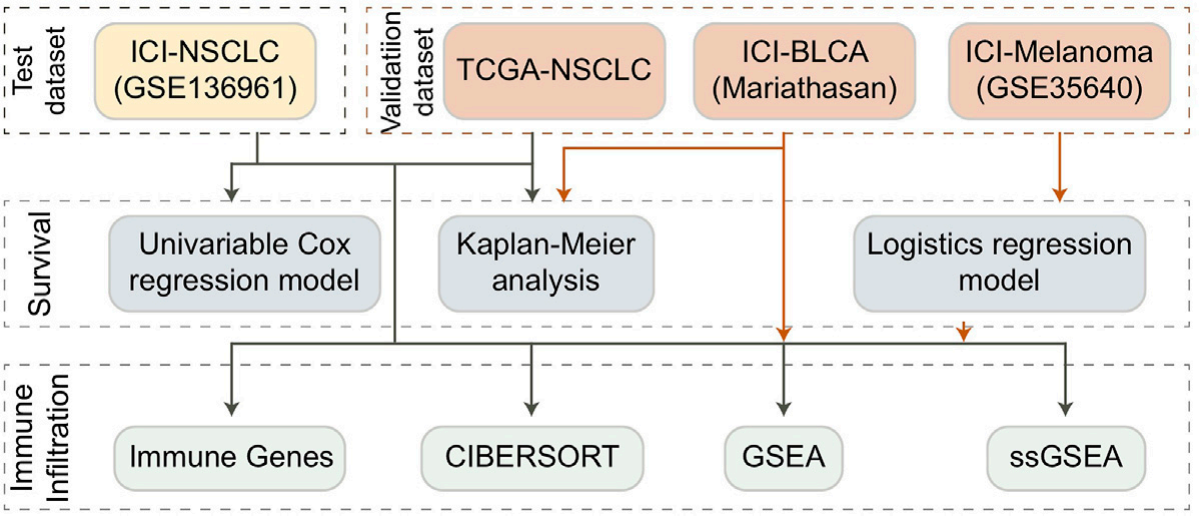
Flowchart of data processing for the TCGA dataset and ICI-treated cohort.

**FIGURE 2:**
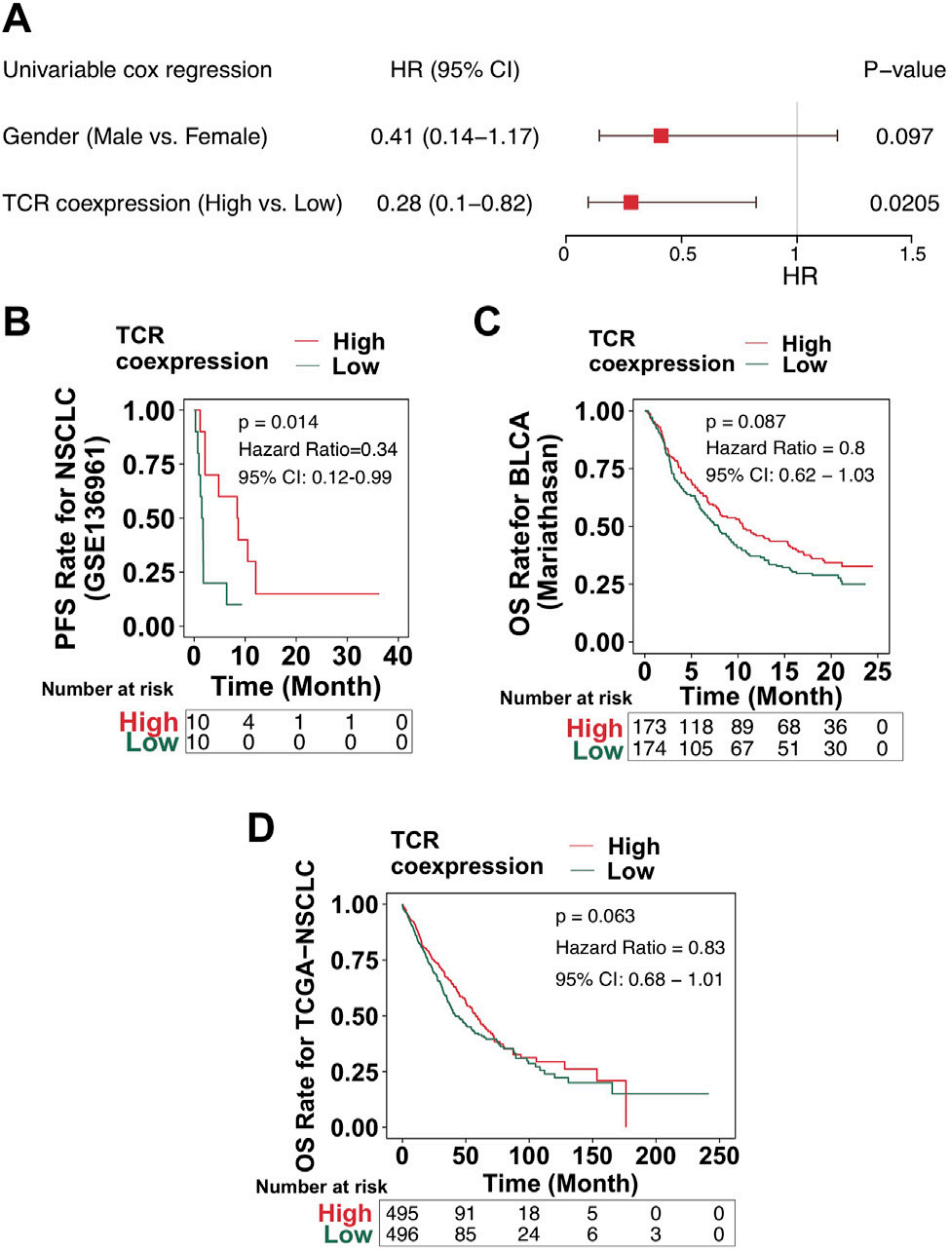
High TCR coexpression signature was associated with improved prognosis of patients receiving immunotherapy. **(A)** The results of the univariate regression analyses displayed as a forest map (GSE136961). The main part of the forest map is used to show the HR and 95% confidence intervals. Factors associated with improved prognosis are log10(HR) < 1, and those associated with poor prognosis are log10(HR) > 1. **(B)** KM survival curves of PFS for NSCLC patients from the ICI cohort (GSE136961). **(C)** KM survival curves of OS for patients in the ICI-BLCA cohort (Mariathasan et al.). **(D)** KM survival curves of OS for patients in the TCGA-NSCLC cohort.

Our results showed a positive relationship between the TCR coexpression signature and the survival benefit and immune response of NSCLC patients treated with ICB. The process used to analyze our data is shown in detail in Figure 1. Firstly, we collected from a public database the expression data for an NSCLC cohort that had received immunotherapy and used it to calculate the TCR coexpression signature of each patient. For the ICI-NSCLC cohort, univariate Cox regression analysis showed that the TCR coxpression signature can be used as a predictor of clinical prognosis for NSCLC patients receiving immunotherapy [Figure 2A; *p* = 0.0205; Hazard ratio (HR) = 0.41]. Further analysis showed that the gender of patients was not related to the prognosis of NSCLC patients receiving immunotherapy (Figure 2A). No other clinical features of this cohort were available for analysis. The results of a survival rate analysis showed that the NSCLC group with a high TCR coexpression signature had significantly improved progression-free survival (PFS) (log-rank *p* = 0.014; HR = 0.34; 95%Cl: 0.12–0.99; Figure 2B). In the ICI-BLCA (Mariathasan et al.), we found that patients with a high TCR coexpression signature tended towards a prolonged PFS, although the results were not statistically significant (*p* = 0.087; HR = 0.8; Figure 2C). It should be noted that, although *p* > 0.05, the sample size of this cohort was small and thus may not be representative. To clarify the relationship between the TCR coexpression signature and clinical prognosis of NSCLC patients receiving routine treatment, the TCGA-NSCLC cohort was used for subsequent analysis. In the TCGA-NSCLC cohort (Figure 2D), the TCR coexpression signature did not show a significant relationship with the survival time of patients undergoing routine treatment (log-rank *p* > 0.05). The above results suggest that TCR coexpression signatures may be a suitable biomarker for predicting the treatment response of NSCLC patients receiving ICB therapy.

### High TCR Coexpression Signatures Were Related to a High Infiltration of Activated Immune Cells

**FIGURE 3:**
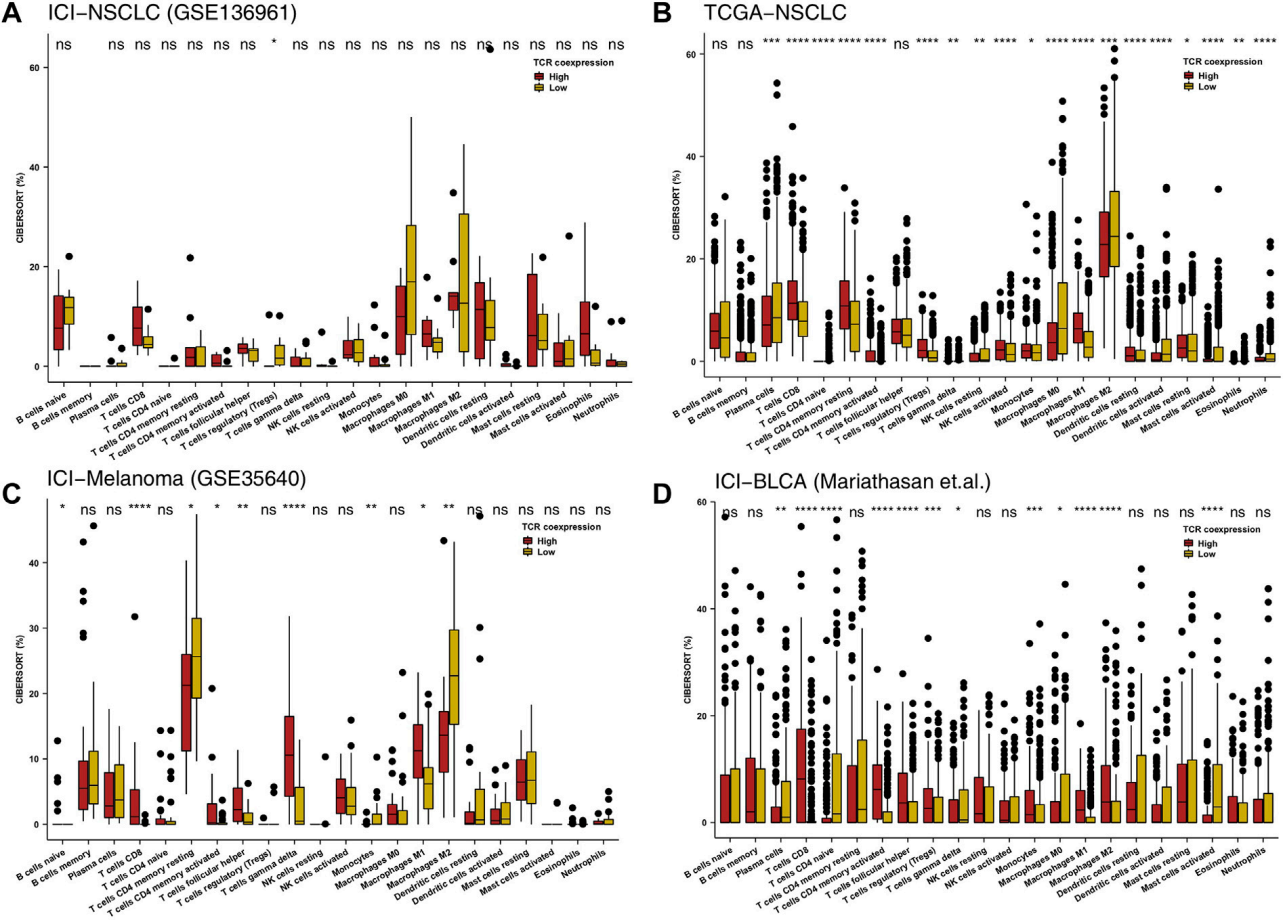
High TCR coexpression signature was associated with a high proportion of activated immune cells. The 22 immune cell types estimated by CIBERSORT methods between the high- and low- TCR coexpression signature groups of the ICI-NSCLC **(A)**, TCGA-NSCLC **(B)**, ICI-Melanoma (GSE35640) **(C)**, and ICI-BLCA (Mariathasan et al.) **(D)** cohorts. The range of *p*-values is presented by the asterisks above each box plot (*p < 0.05; **p < 0.01; ***p < 0.001; ****p < 0.0001; Mann-Whitney U test).

Immune cells play an important role in detecting and killing tumor cells in the TIME. To clarify the relationship between a high TCR coexpression signature and the prognosis of immunotherapy, we used the CIBERSORT algorithm and evaluated the abundance of immune cell infiltration in the TIME. In the ICI-NSCLC cohort, we found that the TIME of the high TCR coexpression signature group had significantly less regulatory T lymphocytes when compared to low TCR coexpression signature group (*p* < 0.05; Figure 3A). In the TCGA-NSCLC cohort, we found that the high TCR coexpression signature group had a high infiltration of CD8+T cells, activated memory CD4+T cells, activated NK cells, and m1-type macrophages in the TIME. Additionally, the degree of infiltration for some immune cells with suppressed or static function in the high group was significantly lower than that in the low group. This included naive CD4+T cells, gamma delta T cells (γδ T cells), resting NK cells, and resting mast cells (*p* < 0.05; Figure 3B). In the ICI cohort (GSE35640), CD8+T cells, activated memory CD4+T cells, follicular helper T cells (TFH), γ δ T cells, and M1-type macrophages were significantly more frequent in patients with a high TCR coexpression signature. We also found that the relative abundance of CD4+T cells and M2-type macrophages in the high group was significantly lower than that in the low group (*p* < 0.05; Figure 3C). For the second ICI cohort (Mariathasan et al.), the high TCR coexpression signature group showed increased CD8+T cells, activated memory CD4+T cells, TFH, and M1-type macrophages in the TIME. Accordingly, the high group had a lower proportion of resting memory CD4^+^ T cells and M0-type macrophages in the TIME (*p* < 0.05; Figure 3D). The above results suggest that a high TCR coexpression signature is related to a high infiltration of activated immune cells.

### High TCR Coexpression Signatures Were Related to High Expression Levels of Anti-Tumor Related Immune Genes

**FIGURE 4:**
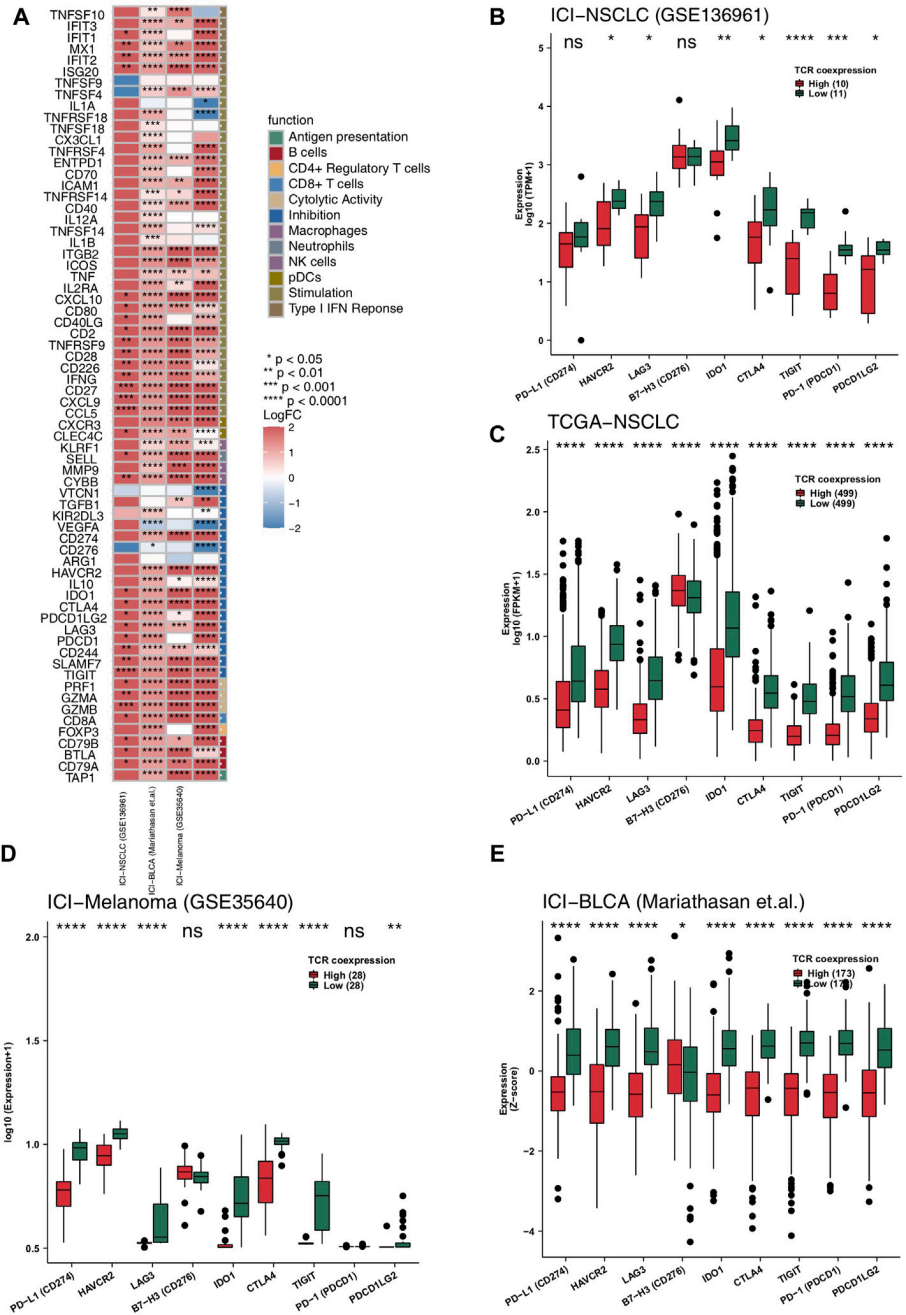
High TCR coexpression signature was associated with high expression levels of immune-related genes. **(A)** Comparison of the expression levels of immune-related genes between the high- and low- TCR coexpression signature groups. Heat map depicting the mean differences in immune-related gene mRNA expression between high- and low- TCR coexpression signature groups across different cohorts. The *x*-axis of the heat map indicates different cohorts, and the *y*-axis indicates gene names. Each square represents the fold change or difference of each indicated immune-related gene between the high- and low- TCR coexpression signature groups in each cohort. Red indicates up-regulation and blue indicates down-regulation. Box plots comparing the expression levels of immune checkpoint molecules between the high- and low- TCR coexpression signature groups from the ICI-NSCLC **(B)**, TCGA-NSCLC **(C)**, ICI-Melanoma (GSE35640) **(D)**, and ICI-BLCA (Mariathasan et al.) **(E)** cohorts. The range of p-values is presented by the asterisks above each box plot (*p < 0.05; **p < 0.01; ***p < 0.001; ****p < 0.0001; Mann-Whitney U test).

Anti-tumor related immune genes include those relating to cytotoxic T lymphocytes, antigen processing and presentation, and immune stimulation. We put together a list of relevant anti-tumor immunity genes and analyzed them on-by-one in the four data sets of this study (Figure 4A). The heatmap in Figure 4A shows multiple changes in expression level for these genes in both the high and the low TCR coexpression groups. It can be seen from this figure that the expression levels of many cytotoxicity related genes (CD8A, GZMB, GZMA, and PRF1), chemokines (CXCR3, CCL5, CXCL9, and CXCL10), inflammatory cytokines (INFG, IL1, TNFSF4, and TNFSF9), and antigen processing and presentation related genes (TAP1) in the high TCR coexpression group were significantly higher than those in low group. We then analyzed the differences in common immune checkpoint molecules between the two groups. In the ICI-NSCLC cohort (GSE136961), compared with the low TCR coexpression group, the high group shows a significantly lower expression of immune checkpoint molecules, such as HAVCR2, LAG3, IDO1, CTLA4, TIGIT,PD-1, and PDCD1LG2 (Figure 4B). In the TCGA-NSCLC cohort, except for B7-H3, the expression of remaining checkpoint molecules in the high group was also significantly lower than that in low group (*p* < 0.05; Figure 4C). The other two cohorts undergoing immunotherapy showed similar results, with the expression of most checkpoint genes in the low TCR coexpression groups being significantly higher than in the high groups (Figures 4D, E).

### High TCR Coexpression Signature is Related to High Activity of Anti-Tumor Related Signal Pathways

**FIGURE 5:**
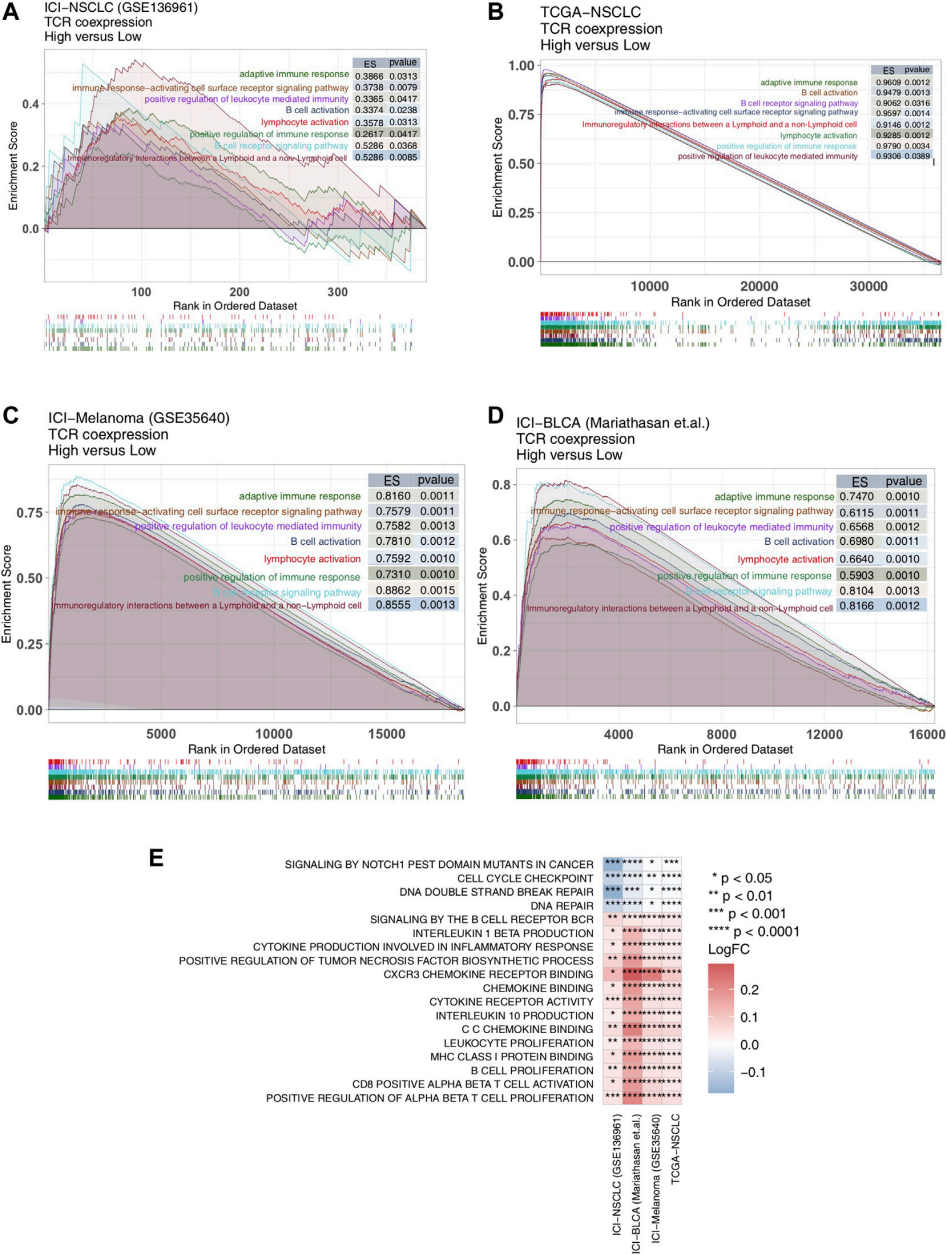
High TCR coexpression signature was associated with a high degree of activated immune-related signaling pathways. Results of the GSEA for the ICI-NSCLC **(A)**, TCGA-NSCLC **(B)**, ICI-Melanoma (GSE35640) **(C)**, and ICI-BLCA (Mariathasan et al.) **(D)** cohorts. The low TCR coexpression signature group served as the control group. Enrichment score (ES) > 0 indicates that the corresponding pathways were significantly enriched in the experimental groups (high TCR coexpression signature group). The color of the curves corresponds to the font colors of the pathway names. **(E)** Heat map depicting the mean differences in the ssGSEA score of signaling pathways between high- and low- TCR coexpression signature groups across different cohorts. The *x*-axis of the heat map indicates different cohorts, and the *y*-axis indicates signaling pathways. Each square represents the fold change or difference of each indicated ssGSEA score of signaling pathways between high- and low- TCR coexpression signature groups in each cohort. Red indicates up-regulation; blue indicates down-regulation.

Signaling pathways also play an important role in anti-tumor activity, so we evaluated them in our patient cohorts using GSEA and ssGSEA. We found significant up-regulation of anti-tumor immune-related signal pathway activity [Enrichment score (ES) > 0; *p* < 0.05] in the high TCR coexpression signature group (Figures 5A–D). This included the B cell receptor signaling pathway, adaptive immune response, B cell activation, immune response-activating cell surface receptor signaling pathway, positive regulation of immune response, lymphocyte activation, positive regulation of leukocyte mediated immunity, and immunoregulatory interactions between lymphoid and a non-lymphoid cells. We utilized the ssGSEA algorithm to evaluate the activity of each pathway for every patient and found, in all four cohorts (Figure 5E), activation of CD8^+^ T cells, proliferation of B cells, and lymphocytes, binding of chemokines including CXCR3, and production of cytokines (such as IL-10,IL-1). Also, the cytokine-mediated inflammatory response pathway showed significantly more activation in the high TCR coexpression group when compared to the low group. At the same time, activation of cell cycle checkpoint and DNA damage repair signal pathways were significantly lower in the high group.

## Discussion

In this study, we found that the TCR coexpression signature may be used as a biomarker to predict the prognosis of immunotherapy for NSCLC, with a high signature indicating a better prognosis. In addition, our results revealed that patients with a high TCR coexpression signature have a TIME with anti-tumor characteristics, such as a higher proportion of functional activated immune cells, lower proportion of functional depleted immune cells, and high expression of cytotoxicity, antigen treatment and presentation, genes related to immunostimulation, and a highly activated anti-tumor related immune response pathway.

As patients with a high TCR coexpression signature had a consistently higher proportion of functional activated immune cells, we suggest the metric as one way to significantly improve the prognosis of immunotherapy. The immune system response to tumors is extremely complex, and the new antigen polypeptides formed by tumor mutations need to be both effectively presented by HLA-I and recognized by T lymphocytes carrying specific TCR. This is the key to immune activation. Previous studies have shown, in tissue samples of patients with advanced melanoma, that the degree of CD8^+^ T cell infiltration can adequately predict the efficacy of PD-1/PD-L1 monoclonal antibody treatment (Tumeh et al., 2014). Antigen treatment and presentation are also very important components of the anti-tumor immune response (Wang et al., 2019; Yi et al., 2022), with previous studies showing that antigen treatment and signature presentation are related to a better prognosis for patients undergoing immunotherapy (Wang et al., 2019). In the process of antigen presentation, TAP-mediated peptides are transported into the endoplasmic reticulum cavity, where they combine with MHC-I complex, finally resulting in T cells which recognize new cell surface antigens (Schumacher and Schreiber, 2015). It has been found that M1 macrophages are able to use two different mechanisms simultaneously to destroy tumor cells once they have been recognized (Hu et al., 2016; Liang et al., 2020). One is that M1 macrophages directly mediate cytotoxicity in order to kill tumor cells. The other is that, stimulated by IFN-γ, macrophages can increase the secretion of inducible nitric oxide synthase, cell adhesion molecules, and other substances which enhance their tumor killing effect (Garrido-Martin et al., 2020). M2 macrophages are able to promote the proliferation of tumor cells through the arginase pathway, and can also participate in tumor angiogenesis (Garrido-Martin et al., 2020). For example, they can produce urokinase-type plasminogen activator and induce the formation of capillary networks via the release of various matrix metalloproteinases (Jayasingam et al., 2019). Additionally, M2 macrophages destroy the basement membrane of endothelial cells by secreting serine protease, metalloprotease, and cathepsin, and are able to decompose a series of collagen and other components of the extracellular matrix. In this way, M2 macrophages help the migration of tumor interstitial cells and tumor cells (Jayasingam et al., 2019; Ham et al., 2020). Using CIBERSORT, a calculation method for inferring leukocyte subtypes from tumor expression data, we found that M2 type macrophages were more predominant than M1 type macrophages in patients with low TCR coexpression signature (M2 type TAM predominant). According to the above results, we determined that patients with a low TCR coexpression signature were more likely to show TIME factors that promote the polarization of macrophages from M1 to M2 type, while in those with high TCR coexpression signatures, factors that maintain the polarization of M1 macrophages and encourage CD8^+^ T cell infiltration were more dominant. In addition, the results of the GSEA and ssGSEA showed that the activity of signal pathways such as lymphocyte activation and proliferation were significantly up-regulated in patients with a higher TCR coexpression signature.

Besides immune cells, high levels of inflammatory cytokine expression and highly activated inflammatory cytokine signaling pathways have also been suggested as mechanisms by which the high TCR coexpression signature group significantly improves the prognosis of immunotherapy. Cytokines, such as interleukins, also play an important role in the TIME. Interleukins are the lymphatic factor of interaction between leukocytes or immune cells, and are essential for transmitting cellular information. They activate and regulate immune cells, mediate the activation, proliferation, and differentiation of T and B cells, and also play an important role in the inflammatory reaction. Ayers and his colleagues (Cristescu et al., 2018) defined a GEP of T cell inflammation as containing IFN-γ response genes, antigen presentation, chemokine expression, cytotoxicity, and adaptive immune resistance. They found that patients with higher GEP scores of T cells were more likely to benefit from immunotherapy. In addition, Cristescu et al. found that patients with high TMB and GEP expression undergoing treatment with pabrizumab had significantly improved PFS compared to patients with low TMB or GEP expression. In our study, we found that genes related to cytotoxicity (CD8A, GZMB, GZMA, and PRF1), chemokines (CXCR3, CCL5, CXCL9, and CXCL10), inflammatory cytokines (INFG, IL1, TNFSF4, and TNFSF9), and antigen processing and presentation related genes (TAP1) were significantly up-regulated in patients with a high TCR coexpression signature. In addition, the results of our GSEA and ssGSEA showed that the high TCR coexpression signature group had a higher degree of chemokine binding, CXCR3 chemokine binding, cytokine (such as IL-10, IL-1) production, cytokine-mediated inflammatory response, and other signal pathways.

Although our results are promising, some limitations in this study exist. Firstly, NSCLC cohorts with both immunotherapy prognosis data and expression data are very rare, which resulted in the use of only one NSCLC cohort collected from the public database, and the use of other cancer immunotherapy cohorts (such as melanoma and urinary system tumors) for the follow-up verification of TCR coexpression. Therefore, for future research we will continue to collect data for NSCLC patients receiving immunotherapy, to further verify the relationship between TCR coexpression and predicted prognosis. Secondly, only ssGSEA was used to estimate TCR coexpression. Moreover, the relationship between TCR diversity (such as the number of clonal species) and the TCR coexpression signature is still not well established. Considering these limitations, we were not able to comprehensively explore the potential mechanism between TCR coexpression signatures and the prognosis of NSCLC patients receiving immunotherapy.

## Conclusion

In this study, we found that a high TCR coexpression signature is a potential biomarker for the prognosis of NSCLC in patients treated with ICB. With regard to the TIME, we found that patients with a high TCR coexpression signature have an immune microenvironment which promotes anti-tumor activity.

## Data Availability

The original contributions presented in the study are included in the article/Supplementary Material, further inquiries can be directed to the corresponding authors.
